# Effect of Suprachoroidal Triamcinolone Acetonide on Intraocular Pressure in Macular Edema: A Retrospective Study

**DOI:** 10.7759/cureus.77282

**Published:** 2025-01-11

**Authors:** Ali Nauman, Kashif Iqbal, Mishal Seyal

**Affiliations:** 1 Ophthalmology, LRBT (Layton Rahmatulla Benevolent Trust) Free Eye Hospital, Lahore, PAK; 2 Vitreoretinal Ophthalmology, LRBT (Layton Rahmatulla Benevolent Trust) Free Eye Hospital, Lahore, PAK

**Keywords:** iop, macular edema, steroid, suprachoroidal, triamcinolone acetonide

## Abstract

Introduction

Macular edema causes decreased vision in diseases like diabetic retinopathy, uveitis, retinal vein occlusions and post-cataract surgery cystoid macular edema. Steroids in the depot form of triamcinolone acetonide (TA) increase the duration of action, but due to a number of complications, especially raising intraocular pressure (IOP), anti-vascular endothelial growth factor (anti-VEGF) injections are now considered the mainstay of treatment. The suprachoroidal space provides an alternate route for steroid delivery, limiting the drug to the posterior segment, hence preventing the adverse effects on the anterior segment. This study aimed to determine the safety of suprachoroidal steroids with respect to their effect on IOP.

Methods

This retrospective study involved patients who received suprachoroidal TA injections for macular edema: diabetic retinopathy, retinal vein occlusions, uveitis and cystoid macular edema post-cataract surgery at Layton Rahmatulla Benevolent Trust (LRBT) Free Eye Hospital, Lahore, Pakistan. Manual medical records from two years were accessed and all patients were included in the study. Patients with a history of ocular hypertension, glaucoma and steroid responsiveness had not received the injection. Crystalline steroid particles of 4 mg/0.1 mL, using an intravenous TA (K-Kort; GlaxoSmithKline, Brentford, UK), were injected into the suprachoroidal space through a 30-gauge syringe with a custom plastic sleeve from a 24-gauge branula exposing 0.1 mm of the bevel. IOP was recorded at baseline before the injection and at weeks 2, 4 and 8. Repeated measures multivariate analysis of variance (ANOVA) was used to compare IOP measurements at the different time intervals.

Results

A total of 61 patients were included, with 70 eyes being assessed, at a mean age of 54.2 ± 10.4 years. Baseline mean IOP was 16.41 ± 2.62 mmHg, 17.04 ± 3.09 mmHg at week 2, 16.30 ± 2.95 mmHg at week 4 and 15.73 ± 1.83 mmHg at week 8. Between baseline IOP and week 2, the mean difference was 1.11 ± 0.66 mmHg (p = 0.59), 0.26 ± 0.43 mmHg from week 2 to week 4 (p = 1.00), and 1.36 ± 0.51 mmHg from week 4 to week 8 (p = 0.51). The mean IOP decreased by 0.50 ± 0.53 mmHg (p = 1.00) from baseline by eight weeks. The differences between IOP, different causes of macular edema and age or gender were not statistically significant. Two patients (1.4%) had a temporary rise in IOP above 24 mmHg requiring ocular medication, with one having a rise above 30 mmHg.

Conclusion

A single injection of suprachoroidal TA temporarily raises the mean IOP, but the increase is insignificant and settles to baseline by two months. Further studies would be required to establish suprachoroidal triamcinolone as a cost-effective and safe treatment for macular edema.

## Introduction

Macular edema is the most common cause of decreased vision due to retinal pathology [1]. At a cellular level in the neurosensory retina, the breakdown of the blood-retinal barrier causes the accumulation of fluid and inflammatory mediators such as the vascular endothelial growth factor (VEGF) in the extracellular spaces between the photoreceptors and neuronal cells which then interferes with neuronal transmission [2]. This is what causes the decrease in visual acuity in macular edema. It can be due to a variety of causes including diabetic retinopathy, uveitis, retinal vein occlusions or post-cataract surgery cystoid macular edema.

Steroids have been widely used for macular edema through various routes, including periocular injection, intravitreal injection or intravitreal implant [3,4]. Injection in the depot form of a synthetic steroid crystal, such as triamcinolone acetonide (TA), or a slow-release implantable device into the vitreous, such as dexamethasone or fluocinolone acetonide implants, tends to increase the duration of action and has proven to be effective in reducing macular edema [5-9]. Anti-vascular endothelial growth factor (anti-VEGF) drugs have now become the mainstay of treatment for macular edema in diabetic retinopathy and retinal vein occlusions [10,11]. Still, newer anti-VEGF drugs with a wider profile of action and greater efficacy are being developed, causing steroids to become redundant in their use for macular edema. With the abundance of anti-VEGF drugs available to the ophthalmologist, steroids seem to be a step backward in the treatment of macular edema.

In underdeveloped populations, intravitreal steroids are still in use for macular edema because of their cost-effectiveness in comparison to anti-VEGF drugs. Dexamethasone, TA and fluocinolone acetonide all have a half-life of two to three hours in the vitreous [12]. Whether injections or implants, they are not without adverse effects, including cataracts and glaucoma [13], in addition to the complications of an intravitreal injection, such as lens penetration, endophthalmitis, retinal pigment epithelial tear or retinal detachment [14].

Alternate, less invasive routes present us with an opportunity to deliver the steroids without incurring the aforementioned adverse effects. Steroid injections into the suprachoroidal space have emerged as such a route [15]. The suprachoroidal space is a potential space of about 35 µm [16], but this has been largely reserved for resistant cases of macular edema since there are certain concerns about its safety, particularly a rise in intraocular pressure (IOP). This provides a position through which a depot steroid can be placed in the eye with minimal exposure of the drug to the anterior segment, preventing the complications discussed earlier.

This study seeks to evaluate the effect of suprachoroidal TA on IOP over two months of follow-up and to determine if a significant rise in IOP is noted. This is a valid concern that needs to be addressed if this route is to be considered a safer and more effective alternative to intravitreal steroids and perhaps even anti-VEGF injections.

## Materials and methods

A retrospective, non-randomized study was carried out involving patients who had undergone suprachoroidal TA injections at Layton Rahmatulla Benevolent Trust (LRBT) Free Eye Hospital, Lahore, Pakistan. Approval was acquired from the Hospital Ethical Review Board. Manual medical records from the past two years, from January 1, 2022, to January 1, 2024, were accessed, and all patients were included in the study. These patients had received a single injection in one or both eyes for four common causes of macular edema, namely diabetic retinopathy, retinal vein occlusions, uveitis and cystoid macular edema post-cataract surgery. Patients with a history of ocular hypertension, at risk for or a history of glaucoma, and a history of steroid responsiveness had not received the injection. Both male and female patients were included with no age limit, and patients who had failed to complete the follow-up visits required to observe the effect of IOP were omitted.

A commercially available intravenous aqueous suspension of 40 mg/mL TA (K-Kort; GlaxoSmithKline, Brentford, UK) was used to prepare crystalline particles of 4 mg/0.1 mL TA, which had been separated using a Millipore filter (Millipore Corp, Bedford, MA, USA). These steroid crystals had been injected into the suprachoroidal space at a distance of 4 mm from the corneal limbus using a 30-gauge syringe (BD Insulin Syringe with BD Ultrafine Needle; Becton, Dickinson and Company, Franklin Lakes, NJ, USA) along with a custom-made plastic sleeve from a 24-gauge cannula, exposing only the bevel (about 0.1 mm), to only enter the suprachoroidal space and prevent an inadvertent intravitreal injection. Anterior chamber paracentesis had then been carried out in all the patients to prevent an immediate spike in IOP.

IOP recorded at baseline prior to the injection and on subsequent follow-up visits at weeks 2, 4 and 8 was documented, along with the age of the patients and the cause of macular edema. Statistical analysis was carried out using IBM SPSS Statistics for Windows, Version 25 (Released 2017; IBM Corp., Armonk, NY, USA), repeated measures multivariate analysis of variance (ANOVA), to compare IOP measurement at the different time intervals. A p-value less than 0.05 was considered significant.

## Results

A total of 61 patients were included in the study, with 70 eyes being assessed. There were 33 males (54%) and 28 females (46%), with a mean age of 54.2 ± 10.4 years. The causes of macular edema identified are given in Table 1.

**Table 1:** Cause of macular edema (n = 70 eyes) DME, Diabetic macular edema; CRVO, Central retinal vein occlusion; BRVO, Branch retinal vein occlusion; CME, Cystoid macular edema

Cause	No. of eyes
DME	49
CRVO	3
BRVO	9
Uveitis	5
Post cataract surgery CME (Irvine Gass syndrome)	4

The mean IOP recorded at baseline was found to be 16.41 ± 2.62 mmHg, 17.04 ± 3.09 mmHg at week 2, 16.30 ± 2.95 mmHg at week 4 and 15.73 ± 1.83 mmHg at week 8, as shown in Table 2, with a p-value of 0.025 significant for a change in mean IOP over time.

**Table 2:** Mean IOP IOP, Intraocular pressure

Duration	Mean IOP (mmHg)
Baseline	16.41 ± 2.62
Week 2	17.04 ± 3.09
Week 4	16.30 ± 2.95
Week 8	15.73 ± 1.83

A comparison was also carried out between the mean baseline IOP measurement and that from week 2; the difference was 1.11 ± 0.66 mmHg (p = 0.59), 0.26 ± 0.43 mmHg between week 2 and week 4 (p = 1.00) and 1.36 ± 0.51 mmHg between week 4 and week 8 (p = 0.51), as depicted in Table 3.

**Table 3:** Comparison of mean IOP ^a^Statistical adjustment for pairwise comparison with Bonferroni correction IOP, Intraocular pressure

Duration	Difference in mean IOP (mmHg)	p-value^a^
Baseline - week 2	1.11 ± 0.66	0.59
Week 2 - week 4	0.26 ± 0.43	1.00
Week 4 - week 8	-1.36 ± 0.51	0.51

By eight weeks, there was a decrease in mean IOP from baseline of 0.50 ± 0.53 mmHg (p = 1.00). There were no statistically significant differences between changes in IOP and age or gender. Post hoc tests did not prove any significant difference in change of IOP between the different causes of macular edema that were studied. Only two patients (1.4%) had a temporary rise in IOP above 24 mmHg requiring ocular medication to control it. Only one of these had a rise above 30 mmHg. There was found to be no evidence of an accidental intravitreal injection or major adverse events such as acceleration of cataract formation or endophthalmitis.

## Discussion

Despite the success of anti-VEGF injections in treating macular edema, steroids still have a role to play. Intravitreal steroid injections and implants have consistently been shown to cause ocular hypertension among other complications. Suprachoroidal TA has garnered evidence as being effective for the treatment of macular edema. Its safety, especially the risk of ocular hypertension, remains a concern.

This retrospective study attempts to establish the safety of suprachoroidal steroids with respect to their effect on IOP. Data from 70 eyes of 61 patients were evaluated with repeated measurements of IOP over two months (eight weeks). The mean IOP at baseline was 16.41 ± 2.62 mmHg, increasing to 17.04 ± 3.09 mmHg at week 2, followed by a progressive decline to 16.30 ± 2.95 mmHg at week 4 and 15.73 ± 1.83 mmHg by week 8, with the p-value of 0.025 being statistically significant for a change in mean IOP. The mean IOP decreased from baseline to eight weeks by 0.50 ± 0.53 mmHg (p = 1.00). Two patients out of the 61 (1.4%) required ocular medication for control of IOP, eventually returning to normal without glaucoma therapy. Both of these patients had received the injection for diabetic macular edema (DME).

Previous studies on the efficacy and safety of suprachoroidal TA, such as the HULK trial [17] for DME, included a treatment-naïve group that received both intravitreal aflibercept and suprachoroidal TA injection and a previously treated group that received only suprachoroidal TA injection. Mean IOP was reported as 13.8 mmHg at baseline, and it was 14.2 mmHg at six months. IOP increased in two patients. A study for refractory DME (TYBEE study) [18] included a concomitant active group who received both suprachoroidal TA and aflibercept at baseline and 12 weeks. In these patients, IOP increased by 1.6 mmHg above baseline by week 4 and then stabilized. IOP of more than 30 mmHg occurred in three patients, and a rise of >10 mmHg occurred in five patients. These studies included patients with concomitant intravitreal aflibercept and suprachoroidal TA injections, which would have caused a greater rise in IOP compared to our patient results. Another study by Tayyab et al. [19] involving patients with resistant DME in the South Asian population reported a mean IOP at baseline of 13.37 ± 2.81 mmHg and 13.45 ± 2.32 mmHg at three months. One patient was reported to have an increase in IOP requiring glaucoma medication.

The Tanzanite study for macular edema in retinal vein occlusion included patients who received a combination of intravitreal aflibercept and suprachoroidal TA injection [20]. Increased IOP was reported in four patients (17.4%). These findings are consistent with our results in that the increase in IOP is statistically insignificant and transient, but these studies had a small sample size and concomitantly used intravitreal aflibercept and suprachoroidal TA.

Previously reported safety of suprachoroidal TA for macular edema in non-infectious uveitis, as evaluated in the AZALEA trial, reported a mean IOP of 13.3 mmHg at baseline and 15.2 mmHg at week 24 in the study eye [21]. Six (15.8%) participants had an IOP rise >10 mmHg from baseline and two (5.3%) participants had an IOP >30 mmHg. The PEACHTREE trial also evaluated macular edema in non-infectious uveitis treated by suprachoroidal TA and reported 11.5% with elevated IOP [22].

As for post-cataract surgery cystoid macular edema, in our literature review, we could only find one case series that reported good efficacy and no rise in IOP with the use of suprachoroidal TA [23].

Our current study had its limitations in that it was a retrospective, non-comparative study in which the patient data could only be maintained for two months of follow-up, and there was a lack of racial diversity in the sample population. A randomized case-control trial with a larger, more diverse sample size and a longer follow-up of up to a year would provide more evidence about the safety of suprachoroidal TA.

## Conclusions

Evidence suggests that a single dose of suprachoroidal TA injection is well tolerated. While the mean IOP did increase initially, it eventually decreased to baseline levels by two months after the injection. The increase in IOP was not found to be significant. We recommend further studies to examine the efficacy and safety of suprachoroidal triamcinolone to treat macular edema as a cost-effective and safe alternative to anti-VEGF injections.
